# The utilisation of biliary organoids for biomedical applications

**DOI:** 10.3389/fbioe.2024.1501829

**Published:** 2025-01-07

**Authors:** Zhongwen Lei, Yijun Yang, Yang Xiang

**Affiliations:** ^1^ Department of Hepatobiliary Surgery, Haikou Affiliated Hospital of Central South University Xiangya School of Medicine, Haikou, Hainan, China; ^2^ Haikou Key Laboratory of Clinical Research and Transformation of Digestive Diseases, Haikou Affiliated Hospital of Central South University Xiangya School of Medicine, Haikou, China

**Keywords:** organoids, mechanisms of disease, drug screening, regenerative medicine, tissue engineering

## Abstract

Biliary duct injury, biliary atresia (BA), biliary tract tumors, primary sclerosing cholangitis (PSC), and other diseases are commonly encountered in clinical practice within the digestive system. To gain a better understanding of the pathogenesis and development of these diseases and explore more effective treatment methods, organoid technology has recently garnered significant attention. Organoids are three-dimensional structures derived from stem/progenitor cells that can faithfully mimic the intricate structure and physiological function of tissues or organs *in vitro*. They provide a valuable platform for studying the pathogenesis of biliary tract diseases and offer novel possibilities for repairing and regenerating biliary tract injuries. The main seed cells used to construct biliary tract organoids include primary human biliary tract epithelial cells as well as pluripotent stem cells. The construction of these organoids involves various techniques such as traditional embedding technology, rotary culture technology, hanging drop culture technology, along with emerging approaches like organ chip technology, three-dimensional (3D) printing technology, and four-dimensional (4D) printing technology. This article comprehensively reviews the construction methods of biliary tract organoids while discussing their applications in disease modeling research on disease mechanisms drug screening tissue/organ repair; it also highlights current challenges and suggests future research directions regarding biliary tract organoids which will serve as references for treating common refractory digestive system diseases in clinical practice.

## 1 Introduction

Iatrogenic bile duct injury is a highly significant complication of laparoscopic cholecystectomy, with an incidence ranging from 0.4% to 1.5% (de’Angelis et al., 2021). This can result in the development of biliary strictures, recurrent cholangitis, secondary biliary cirrhosis, end-stage liver disease, and even mortality. Endoscopic Retrograde Cholangiopancreatography (ERCP) biliary stent implantation is a common clinical treatment. However, long-term implantation can lead to cholangitis and re-stenosis, which may require removal after the operation, potentially resulting in secondary injury (Siiki et al., 2018). Liver transplantation serves as a last resort for conditions such as biliary tract injury, BA, and PSC; however, it has limitations due to limited donor availability and autoimmune rejection. In addition, effective treatment options for refractory diseases such as BA, biliary tract tumors, and PSC are currently lacking. To gain a better understanding of the pathogenesis and progression of biliary tract diseases, researchers commonly rely on animal models and two-dimensional (2D) cell models. However, these models often exhibit species differences and individual variations, leading to non-homogeneous research results. Moreover, long-term cultured cells may undergo aging and gene mutations, further compromising the accuracy of experimental findings. In order to explore more effective models for bile duct research, emerging organoids have garnered significant attention in recent years.

**FIGURE 1:**
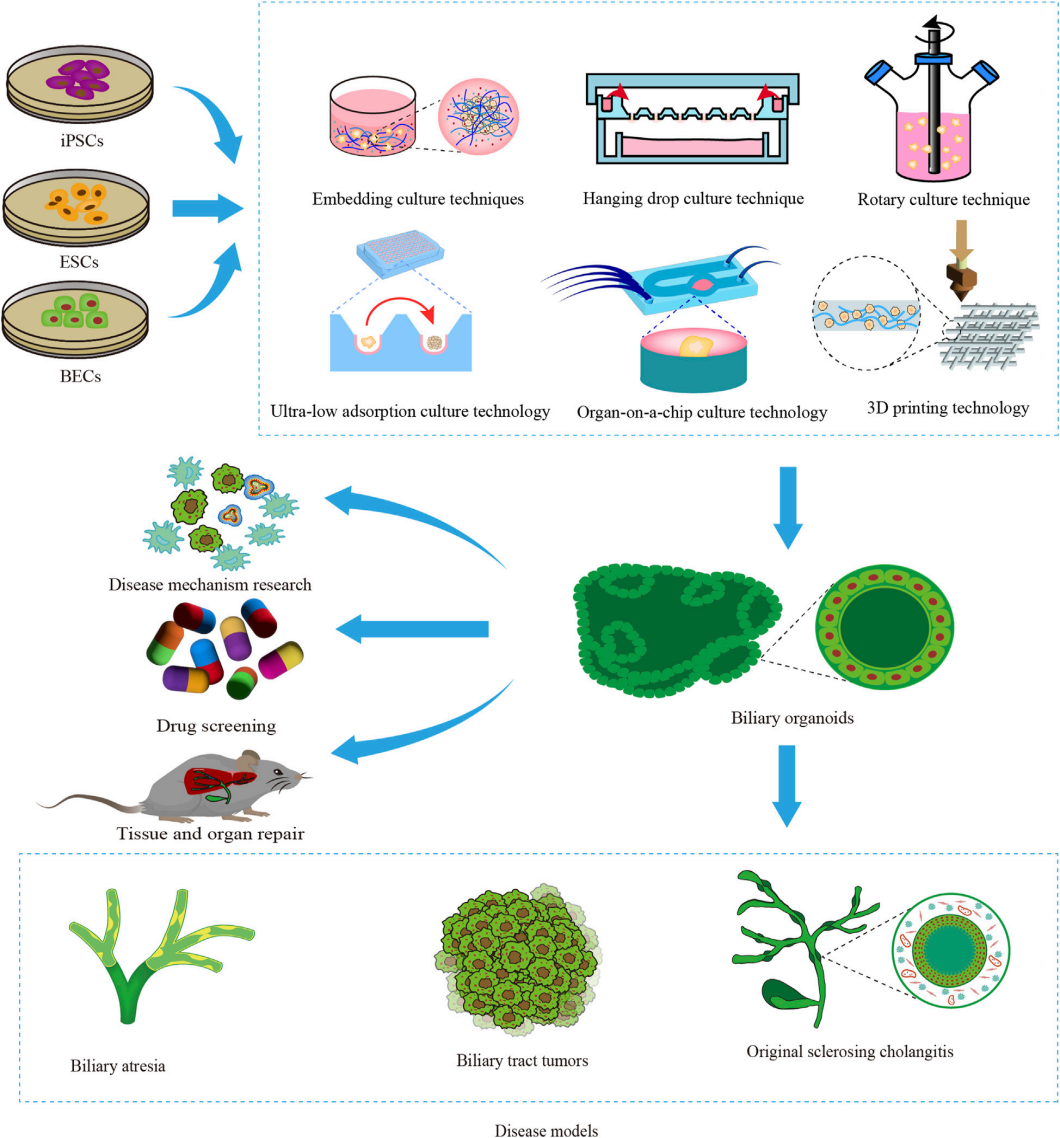
Construction and application of biliary organoids. The diagram provides an overview of the origin of biliary organoid seed cells, including induced pluripotent stem cells (iPSCs), embryonic stem cells (ESCs), and biliary epithelial cells (BECs). The construction of biliary organoids using various techniques has facilitated their extensive utilization in disease modeling, investigation of disease mechanisms, drug screening, and advancement of regenerative medicine.

Due to the origin of seed cells and their highly realistic three-dimensional structure and function, organoid models effectively mitigate the limitations associated with animal models and two-dimensional cell models, thereby exhibiting significant potential in advancing biliary tract research. The development of biliary organoids relies on the inherent capacity of stem/progenitor cells for self-differentiation and self-organization. By utilizing bioreactors such as stirred bioreactor (SBR) and microfluidic bioreactor (MFB), these cells can progressively differentiate into three-dimensional aggregates that closely resemble the morphology and function of natural biliary tracts in an *in vitro* microenvironment, effectively mimicking the growth and development of organs *in vivo*. (Licata et al., 2023; O’Connell and Winter, 2020). This not only offers a novel approach for modeling biliary diseases, exploring underlying mechanisms, and screening drugs but also holds significant potential for regenerating and repairing bile duct injuries (Figure 1). This review provides a comprehensive overview of biliary organoid construction encompassing cell sources, construction techniques while further discussing its biomedical applications, current challenges, and future directions.

## 2 Construction of biliary organoids

Bioreactors and bioactive materials play pivotal roles in promoting the self-renewal, differentiation, and self-organization of stem/progenitor cells to generate 3D aggregates that closely mimic both the morphology and functionality of native biliary tracts, thus facilitating the development of biliary organoids. The construction process of biliary organoids can generally be divided into two main aspects: seed cell selection and construction techniques (Ogoke et al., 2021).

### 2.1 Common seed cells for constructing biliary organoids

The common seed cells that constitute biliary organoids include induced pluripotent stem cells (iPSCs), ESCs, liver progenitor cells (LPCs), and biliary epithelial cells (BECs). BECs possess inherent stem cell potential and can be readily isolated from either biliary tract tissue or liver tissue, which are relatively accessible. Obtaining stem cells, particularly induced pluripotent stem cells (iPSCs), typically involves a complex reprogramming process; however, iPSCs exhibit excellent proliferative capacity and broad differentiation potential (Sampaziotis et al., 2021). Therefore, the choice of seed cell type depends on specific research objectives and application scenarios (Sato et al., 2021).

Roos et al. (2020) successfully generated a biliary organoid using biliary epithelial cells, which exhibited the expression of functional cholangiocyte-related genes (such as ANO1, NKCC1, CFTR, GGT, AQP1) and the basolateral receptor SCTR based on gene array analysis. Additionally, this organoid demonstrated typical cholangiocyte receptors: pancreatopoietin and somatostatin. Elci et al. (2024) developed a stable biliary organoid utilizing biliary epithelial cells that displayed essential characteristics of cholangiocytes including transport activity, primary cilium formation, and protective glycocalyx formation over an extended period. Sampaziotis et al. (2019) constructed distinct biliary organoids using human induced pluripotent stem cells (hiPSCs) and primary biliary epithelial cells, which exhibited expression of biliary markers and stable maintenance of biliary cell function. This approach successfully addressed challenges related to maintaining, promoting proliferation, and achieving large-scale expansion of primary biliary epithelial cells. In2021, Expanding upon their previous research, Sampaziotis et al. (2021) subsequently developed an intrahepatic bile duct repair strategy by implanting a biliary organoid induced from extrahepatic biliary epithelial cells. Their findings have significant implications for the field of regenerative medicine focused on treating intrahepatic bile duct defects.

Wu et al. (2019) employed a liver differentiation medium to induce hepatic differentiation of hiPSCs and successfully established hepatobiliary organoids (HBOs) exhibiting apical-basal polarity and mature biliary cell structures. Immunofluorescence staining targeting cystic fibrosis transmembrane conductance regulator (CFTR) and α-tubulin confirmed the preservation of stable bile duct architecture within HBOs. Additionally, rhodamine 123 (Rh123) staining verified the efficient bile efflux function of these biliary structures. Wang et al. (2019) utilized a novel medium comprising Wnt3A, Activin A, Bone morphogenetic protein (BMP4), and fibroblast growth factor (FGF)2 to induce the differentiation of human embryonic stem cells (hESCs) towards the hepatic specification (HS) stage. Following supplementation with N2, B27, nicotinamide, ghrelin, N-acetylcysteine, epidermal growth factor (EGF), Wnt3A, Wnt enhancer (R-Spondin 1), small compounds A83-01, and forskolin for a duration of 10 days, a robust liver organoid (hEHOs) was generated that effectively maintained the phenotypic characteristics of hepatic stem/progenitor cells. This advancement in differentiating functional hepatocytes and biliary cells has significantly broadened the cell sources available for clinical research purposes.

### 2.2 Construction techniques of organoid

The construction technology of biliary organoids can be divided into traditional construction technology and new construction technology. Traditional construction techniques generally include embedding culture technology, rotary culture technology, hanging drop culture technology, magnetic suspension culture technology, and ultra-low adsorption culture technology, among which the most common one is embedding culture technology. The new construction technologies mainly include organ-on-a-chip technology, three-dimensional printing technology, four-dimensional printing technology, etc.

#### 2.2.1 Traditional construction techniques

The construction techniques for traditional biliary organoids primarily encompass:

##### 2.2.1.1 Embedding culture technology

The term refers to a culture technology wherein cells are encapsulated within a matrix adhesive and subsequently supplemented with various signaling pathway proteins and growth factors, resulting in the formation of an active three-dimensional structure (Habanjar et al., 2021). This method is characterized by its simplicity of operation and mild culture environment; however, the absence of cell-to-cell interactions may pose challenges in achieving cellular aggregation into spheres. Additionally, the high cost of the matrix adhesive and production expenses hinder large-scale implementation (Lee et al., 2021).

Lugli et al. (2016) initially employed the technique of embedding culture to cultivate stem/progenitor cells derived from the gallbladder into a gallbladder organoid, which demonstrated expression of cholangiocyte markers Keratin (KRT)19, cluster of differentiation (CD) 44, and claudin (CLDN)3 and could be maintained *in vitro* for over 1 year. However, when transplanted under the liver capsule, the physiological structure of the organoid could only be sustained for a duration of 2 weeks (Lugli et al., 2016). Zhang et al. (2024) developed a biliary tree stem cells (BTSCs) organoid culture system based on embedding culture technology called BTSCs-expansion-Glycogel-system (BEX-gel system), enabling efficient utilization of limited donor organs such as the gallbladder and bile duct to generate a substantial quantity of BTSCs while facilitating research on *in vivo* transformation of hBTSCs organoids (Zhang et al., 2024). In summary, embedding culture technology is a widely used method for rapidly and conveniently generating biliary organoids. However, the lack of standardization in the matrix gel often leads to compositional variations between batches, resulting in significant heterogeneity among the generated organoids.

##### 2.2.1.2 Rotation culture technique

The rotating cell culture system (RCCS) is commonly employed to continuously rotate the cell culture medium, simulating a microgravity environment for cell growth and facilitating the formation of 3D tissue structures (Mattei et al., 2019). This technology enhances nutrient utilization by cells/tissues and promotes their development; however, it necessitates a high rotation rate. Excessive rotation speed can cause damage to cells and tissues, while insufficient rotation speed may result in tissue/cell sedimentation, thereby limiting growth and development (Ryu et al., 2019).

The research conducted by He et al. (2022) demonstrated the successful construction of a HBOs using liver cells through the technique of rotational cultivation. Initially, liver cells were differentiated into hollow hepatocyte-like organoids (HHOs) using matrix gel embedding technology. Subsequently, these HHOs were placed in a rotating bioreactor with a flowing suspension environment to optimize nutrient absorption and facilitate progenitor cell self-assembly into functional HBOs. As the application of rotational cultivation techniques in the field of biliary tract continues to advance, it is imperative to carefully select appropriate rotational cultivation conditions for constructing biliary tract-like organs, including adjusting rotation speed, optimizing culture medium composition, and incorporating specific growth factors.

##### 2.2.1.3 Hanging drop method

That is, by inverting the cell suspension droplet and using its surface tension and gravity to aggregate cells/tissues into cell spheres at the liquid-eob-gas interface (Sun et al., 2021). This technology can generate a large number of three-dimensional cell spheres of the same size in a short time, which is suitable for industrial production. However, due to the limited volume of droplets, the size of cell spheres produced is relatively small (Zhou et al., 2023).

The hanging drop culture technique facilitates the formation of three-dimensional structures that closely replicate the arrangement and function of biliary cells *in vivo*. However, it is important to note that the intricacies observed *in vivo* may not be fully replicated in terms of extracellular matrix formation and distribution. Furthermore, there might be significant differences in mechanical stress experienced by biliary cells between *in vitro* and *in vivo* environments, potentially impacting the normal physiological characteristics of bile duct-like organs. Therefore, researchers should continuously optimize culture conditions, refine techniques, and explore novel biomaterials and methodologies to enhance both quality and functionality when constructing bile duct-like organs (Ohguro et al., 2024). Currently, this technique primarily finds application within kidney-like or testicular-like organ systems (Parigoris et al., 2022; Yang et al., 2022).

##### 2.2.1.4 Magnetic suspension culture technology

The mixture of magnetic particles/nanoparticles is utilized for cellular magnetization and enhancement of intercellular interaction forces, thereby facilitating cell aggregation into clusters to form cellular spheres (Marques et al., 2022). This technology not only enables control over the geometry of the cellular sphere through magnetic fields but also facilitates co-culturing different types of cells. However, it does not substitute the need for a cell medium and presents challenges in controlling cell size, which limits its applicability (Tepe et al., 2023).

Magnetic levitation cultivation technology can provide a three-dimensional growth environment similar to *in vivo* conditions, enabling contact-free cell growth and minimizing mechanical stress-induced damage between bile duct cells and the culture surface. This promotes the development of intricate bile duct structures (El Harane et al., 2023). However, due to equipment and technical limitations, magnetic levitation cultivation technology is not suitable for long-term cultivation of biliary organs as it lacks efficient removal of metabolic waste and toxins. Nonetheless, this technique has general applicability in salivary gland-like organoids and adipose organoids (Daquinag et al., 2013; Ferreira et al., 2019).

##### 2.2.1.5 Ultra-low adsorption culture technology

The process of forming cell spheres using 96-well plates and 384-well plates, which meet the requirements for high-throughput three-dimensional culture (Xing et al., 2024), is referred to as “exclusion of seed cells through ultra-low adsorption materials, resulting in their aggregation into spherical structures.” This technique is characterized by its operational simplicity and ability to generate large-scale cell spheres with uniform diameters while allowing control over sphere size by adjusting the number of seed cells. However, it still faces challenges associated with a high coefficient of variation (Ryu et al., 2019).

Kim et al. (2023) employed ultra-low attachment culture technology to fabricate a HBOs that possesses both vascular and biliary systems. The vascular system generates perfusable microvessels with lumens, enabling the organoid to mimic liver diseases mediated by interactions between parenchymal and non-parenchymal cells, exhibiting promising applications (Kim et al., 2023). The ultra-low attachment culture technology facilitates co-culture of biliary epithelial cells and mesenchymal stem cells, fostering cell-cell interactions and signaling transduction, recapitulating the arrangement of bile duct cells akin to normal tissue, and forming intricate bile duct-like structures with tremendous potential for application. However, the ability of bile duct epithelial cells to adhere to and receive signals such as growth factors, cytokines, and hormones is limited in long-term culture, which may affect their survival rate and physiological function (Acharya et al., 2024). Moreover, physiological data derived from bile duct organoids generated using ultra-low attachment culture technology diverge from those obtained through alternative culturing methods such as embedding techniques, thereby augmenting the complexity in analyzing diverse outcomes (Acharya et al., 2024).

#### 2.2.2 New construction technology

Traditional cultural technology presents certain drawbacks in the construction of biliary organoids, including a lengthy processing cycle, high costs, and limited morphological design. These limitations can be overcome by employing novel construction methods such as organ-on-a-chip (OOC), 3D, and 4D printing technologies. These advanced techniques enable rapid production of complex organoids with high-throughput and exceptional accuracy (Hockney et al., 2023).

##### 2.2.2.1 Organ-on-a-chip technology

The term OOC refers to the utilization of microfluidic chips for constructing a physiologically relevant microenvironment that encompasses various living cells, functional tissue interfaces, mechanical force stimulation through biological fluids, and more (Deng et al., 2023; Li et al., 2023; Nasiri et al., 2024). This technology enhances mechanical stimulation by integrating biomaterial technology, microfluidic technology, and tissue engineering techniques to precisely regulate multiple system parameters and provide real-time display of diverse functional data associated with tissue and organ function. Consequently, it holds significant application value in the fields of organoid construction, drug screening, and personalized precision medicine (Baptista et al., 2024; Palasantzas et al., 2023). Organoids cultivated within OOC systems exhibit high fidelity in simulating anatomical structures as well as physiological/pathological states of tissues/organs; thus representing a promising culture methodology (Shoji et al., 2023).

The biliary epithelium has been utilized by Du et al. (2020) to fabricate bile duct-like organs with a tubular structure and barrier function through the application of organ chip technology. This innovative organ model provides a robust *in vitro* platform for investigating biliary pathophysiology, allowing independent access to the apical and lateral surfaces of the biliary epithelial cell channels. In 2024, they further advanced their research by integrating a vascular system into the bile duct-like organ using organ chip technology (Du et al., 2023). The biliary epithelial cells within these organs exhibit polarization within the channels, forming mature tight junctions and demonstrating permeability levels comparable to those observed *in vivo* systems. Researchers can investigate distinct reactions between biliary epithelial cells and vascular endothelial cells towards varying flows of perfused blood and bile inside these lined channels. OOC technology can simulate the microenvironment of the biliary tract, including cellular arrangement, fluid dynamics, and physical stimuli. This provides a more physiologically relevant culture environment for biliary organoids. Additionally, it allows for co-culturing different cell types in the biliary tract, facilitating research on interactions and signaling between constituent cells and enhancing the physiological relevance of biliary organoids. The use of organ-chip technology for a complete analysis of specific mechanisms of the biliary tract is good. However, since all human systems are interconnected, the body-on-a-chip concept should be further developed to evaluate the whole system, that is, the interactions between multiple organs, the mechanisms of cell migration, and the cell signaling processes between them (Koyilot et al., 2022).

##### 2.2.2.2 3D/4D printing technology

3D printing technology refers to the utilization of computer-aided design for the fabrication of intricate bioactive tissue/organ structures by printing biocompatible materials, cells, and biomolecules (Kantaros, 2022). This technology offers advantages such as cost-effectiveness, high material utilization rate, a straightforward and convenient process, and customization capabilities for organoids. It is a comprehensive technology with significant personalization potential, offering a high degree of freedom and precision (Assad et al., 2023; Jing et al., 2023). While 3D printing excels in manufacturing static structures, it falls short in simulating the dynamic behavior observed in natural tissues and organs (Mandal and Chatterjee, 2024).On the other hand, 4D printing is an additive manufacturing technique that builds upon 3D printing by incorporating time as the fourth dimension. By subjecting the structure to one or more stimuli, it enables transition into a state of dynamic equilibrium (Kalogeropoulou et al., 2024; Wan et al., 2024). With its ability to fabricate highly complex biological structures effectively addressing some limitations of 3D printing, 4D printing holds great promise for revolutionizing tissue engineering and regenerative medicine (TERM).

Lee et al. (2019) employed 3D printing technology to create a HBOs, using decellularized extracellular matrix (ECM) bioink derived from the liver along with vascular/biliary fluid channels that closely resemble the blood vessels and biliary system. This organ model demonstrates both the functionality of a bile duct system and specific gene expression of the liver, making it a promising platform for *in vitro* drug screening (Lee et al., 2019). The rapid advancement of 3D printing technology has enabled the construction of highly intricate three-dimensional models that closely mirror the structural and physiological characteristics of bile duct organs. These models offer superior cell arrangement and spatial organization, significantly enhancing research accuracy and effectiveness. Moreover, the personalized nature of 3D printing allows for tailored bile duct organ models based on individual patient conditions, which is crucial for disease study and treatment plan development. However, practical applications of 3D printing technology still face challenges such as limited cell growth and differentiation on printed scaffolds due to material compatibility issues that can impact organ development. Furthermore, precise equipment and techniques are required to adjust and control environmental conditions like temperature, pH value, oxygen concentration etc., during 3D cultivation.

The utilization of 4D printing technology presents new prospects for constructing biliary organs, enabling them to adapt and modulate their functionalities in response to external stimuli such as temperature, pH, or humidity variations. This augmentation enhances their physiological relevance. By employing intelligent responsive materials, 4D printing not only mimics the growth and repair processes of biliary ducts but also provides a platform for researchers to observe cellular reactions under diverse conditions, thus generating more realistic biological response models (Chadwick et al., 2020; Li et al., 2024b). However, 4D printing technology imposes relatively high material requirements and necessitates the use of smart materials with precise reaction capabilities to effectively adapt to intricate dynamic changes in biological environments. Simultaneously, it is crucial to enhance the accuracy of 4D printing technology to ensure microstructural consistency. Currently, challenges persist regarding technology and materials when it comes to manufacturing intricate and dynamically responsive biliary organiods. Table 1 presents common techniques employed in organoid construction.

**TABLE 1:** Several common organ construction techniques.

Technology of construction	Type of technology	Advantages	Disadvantages	Type	References
Traditional techniques	Embedding culture techniques	The procedure is simple, demonstrating strong biocompatibility and the ability to control the porosity of the outer wall	The lack of standardization in the matrix, which typically undergoes compositional changes between batches, leads to significant variations in the generated organoids	ICOs, ECOs, GCOs, HBOs	Lee et al., 2021, Lugli et al., 2016, Sampaziotis et al., 2017, Ramli et al., 2020, Amarachintha et al., 2022
	Rotary culture technique	It is capable of simulating the microgravity effect and exhibits a high rate of nutrient utilization	It requires a high rotation rate and expensive equipment	HBOs	Ryu et al., 2019, He et al., 2022
	Hanging drop culture technique	The procedure is simple, the size of the organoids remains consistent, and there is a significant amount of flux	The stability of organoids is suboptimal, and replacing the culture medium presents challenges	Tubuloids,TOs, MOs	Zhou et al., 2023, Parigoris et al., 2022, Yang et al., 2022
	Magnetic suspension culture technology	It facilitates the co-cultivation of multiple cells, enhances cellular interaction forces, and promotes cell aggregation	The equipment is expensive, and controlling the size of cell spheres presents challenges	SGLOs, AOs	Tepe et al., 2023, Ferreira et al., 2019
	Ultra-low adsorption culture technology	It is easy to operate, manageable, and has a high processing capacity	The high coefficient of variation in organoids and the ultra-low adsorption culture plates come at a significant cost	HBOs	Ryu et al., 2019, Kim et al., 2023, Acharya et al., 2024
New technologies	Organ-on-a-chip culture technology	It allows for three-dimensional dynamic cultivation, precise control of physical and chemical stimuli, as well as high-throughput and reliable operations	When certain materials, such as PDMS, are used, the drugs tested in the system exhibit low permeability	BOs	Baptista et al., 2024, Palasantzas et al., 2023, Du et al., 2020 Grant et al., 2021
	3D printing technology	It offers the benefits of personalized customization, a high level of refinement, and rapid modeling speed	The lack of legal frameworks, regulatory measures, and established quality benchmarks is evident	HBOs	Mandal and Chatterjee, 2024, Assad et al., 2023, Jing et al., 2023, Lee et al., 2019
	4D printing technology	The deformation structure can be controlled, the stimulus response types are diverse, and the strain recovery is reversible	The response form is singular, and there is a scarcity of high-performance intelligent materials	GPDOs	Kalogeropoulou et al., 2024, Wan et al., 2024, Chadwick et al., 2020, Li et al., 2024b

Abbreviations: PDMS, polydimethylsiloxane; ICOs, intrahepatic biliary organoids; ECOs, Extrahepatic cholangiocyte organoids; GCOs, gallbladder biliary organoids; HBOs, hepatobiliary organoids; Tubuloids, basal-in and apical-out proximal tubule organoids; TOs, testicular organoids; MOs, mammary organoids; SGLOs, salivary gland-like organoids; Aos, adipose organoids; BOs, biliary organoids; GPDOs, glioblastoma patient derived organoids-like.

## 3 Types of biliary organoids

**FIGURE 2:**
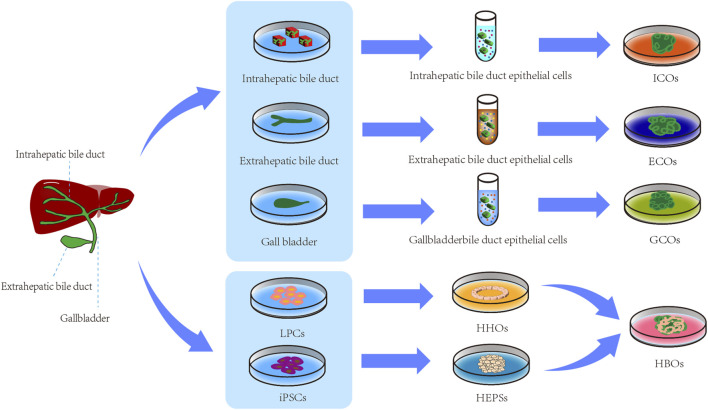
Types of biliary organoids. LPCs, liver progenitor cells; iPSCs, Induced Pluripotent Stem Cells; HHOs, hollow hepatocyte organoids; HEPSs, hepatoblast spheroids; ICOs, intrahepatic biliary organoids; ECOs, extrahepatic biliary organoids; GCOs, gallbladder biliary organoids; HBOs, Hepatobiliary organoids.

The biliary organoids can be named based on their cell of origin, cell type, or anatomical structure. Ary Marsee proposed a reliable nomenclature method that categorizes biliary tract organoids into three types: intrahepatic cholangiocyte organoids (ICOs), Extrahepatic cholangiocyte organoids (ECOs) and gallbladder cholangiocyte organoids (GCOs) (Marsee et al., 2021). The nomenclature proposed by Marsee et al. (2021) provides a systematic framework for classifying and describing biliary organoids, enabling researchers to adopt uniform terminology in their discussions and communications. This standardization significantly reduces confusion in scientific communication and establishes a clear foundation for subsequent research. Building upon the nomenclature of Marsee et al. (2021) the authors introduced HBOs as a category based on the biological characteristics of biliary organoids, providing support for further development of related research. The various types of biliary organoids are illustrated in Figure 2.

### 3.1 Intrahepatic cholangiocyte organoids (ICOs)

Intrahepatic bile duct organoids have significant potential for various applications, including mechanistic research on biliary tract diseases, drug screening, and regenerative medicine (Rodrigues and Banales, 2021). Typically, these organoids are constructed using intrahepatic bile duct epithelial cells. Huch et al. (2015) utilized intrahepatic biliary epithelial cells to generate ICOs that expressed key markers of biliary epithelial cells such as cytokeratin markers KRT19 and KRT7. Moreover, these ICOs could be subcultured in a stable manner, providing a valuable experimental platform for disease modeling, toxicology research, regenerative medicine, and gene therapy targeting the intrahepatic bile duct (Huch et al., 2015). Additionally, hiPSCs also hold potential for constructing intrahepatic bile duct organoids. Carolina et al. (2024) successfully generated ICOs by co-culturing hiPSCs with blood vessels (BVS) composed of immature smooth muscle cells and endothelial cells. These ICOs exhibited characteristics resembling those of bile duct epithelial cells including intercellular junctions, microvilli on the apical membrane, and bile secretion function (Carolina et al., 2024). Transplantation of these ICOs onto the liver surface of cholestatic mice demonstrated their ability to temporarily alleviate cholestasis symptoms while showcasing promising prospects for regenerative medicine in the field of the biliary tract.

### 3.2 Extrahepatic cholangiocyte organoids (ECOs)

Epithelial cells derived from the extrahepatic bile ducts can be expanded and cultured *in vitro* to generate ECOs that exhibit transcriptional and functional characteristics consistent with those of cholangiocytes. Sampaziotis et al. (2017) generated ECOs using the embedding culture technique. They isolated biliary cells from mechanically dissected extrahepatic bile ducts and cultured them with EGF, R-spondin, and Wnt inhibitor (DKK-1). The resulting ECOs exhibited ultrastructural features, including cilia, microvilli, and tight junctions. These organoids expressed biliary markers such as KRT7, KRT19, nuclear hepatocyte factor (NHF) 1β, gamma-glutamyltransferase (GGT), secretin receptor (SCTR), Sodium/cholic acid cotransporter (SLC10A2), CFTR, and SRY-box9 (SOX9). Notably, the expression of SLC10A2 as a biliary marker remains controversial due to its known expression in intestinal epithelial cells of the terminal ileum (Zhou et al., 2014). Moreover, the ECOs could be efficiently cultured within 24 h and expanded through multiple passages while retaining their original properties. This not only provides a novel cell source for biliary disease cell therapy but also offers a new model for studying the extrahepatic bile duct. The study conducted by Verstegen et al. (2020) demonstrated the successful establishment of both ECOs and ICOs. Both types of organs showed similarities in terms of biliary epithelial cell biomarkers, gene expression profiles, proliferative capacity, and functional characteristics. However, unlike ICOs, ECOs did not undergo differentiation into hepatocyte-like cells. This indicates that there are distinct differences in the potential for differentiation between ECOs and ICOs.

### 3.3 Gallbladder cholangiocyte organoids (GCOs)

The gallbladder can serve as a source for isolating stem/progenitor cells, which can be successfully cultured *in vitro* over an extended period of time to generate GCOs that exhibit a remarkably similar structure and partially retain gallbladder functionality. Lugli et al. (2016) employed culture technology to construct GCOs using embedding techniques. They minced the extrahepatic bile duct and gallbladder from 2-month-old mice, incubated them in PBS containing EDTA at 4°C for 2 h to generate a small cell mass before embedding it into Matrigel matrix gel. The resulting GCOs constructs were further cultivated in serum-free medium supplemented with nicotinamide and various growth factors (including EGF, FGF 10, hepatocyte growth factor (HGF), R-spondin-1 and Transcriptional active morphogenetic protein pathway inhibitor (noggin). This led to the final formation of GCOs that expressed gallbladder markers such as CLDN3, epithelial cell adhesion molecule (EPCAM), protruding protein 1, and integrin α6. These GCOs serve as an excellent research model with significant implications for studying liver and biliary tract diseases.

### 3.4 Hepatobiliary organoids (HBOs)

Hepatocytes have the ability to undergo dedifferentiation into LPCs, which can subsequently redifferentiate into both hepatocytes and cholangiocytes during liver development. Therefore, hepatocytes can be utilized for HBOs that contains both hepatocytes and cholangiocytes (Yimlamai et al., 2014). He et al. (2022) developed an HBO using embedding culture technology and rotary culture technology, based on hepatocyte dedifferentiation into LPCs. The resulting HBO expressed NHF4A as a marker for hepatocyte differentiation and KRT19 as a marker for bile duct epithelial cell differentiation, with electron microscopy revealing the presence of both hepatocyte and bile duct structures within the HBO. This platform holds great potential for studying liver development, regenerative medicine, and diseases (He et al., 2022).

Furthermore, various types of stem/progenitor cells including human chemically induced liver progenitor cells (hCLiPs), iPSCs, and hESCs have the potential to differentiate into hepatobiliary organoids. Li et al. (2024a) employed ultra-low adsorption culture technology to establish an hCLiP-derived HBO, which exhibited functional bile ducts, bile duct connections, and mature hepatocytes (MHs). By conducting Rh123 assay and cholyllysyl fluorescein (CLF) dye assay, they demonstrated that this hepatobiliary organoid possessed bile secretion and transport functions, making it a promising model for investigating hepatobiliary diseases, drug screening, and personalized medicine (Li et al., 2024a). Ramli et al. (2020) successfully generated a similar type of HBOs by co-culturing iPSCs and hESCs with matrigel embedding technology. This organoid expressed biomarkers characteristic of both hepatocytes and biliary epithelial cells; normal secretion of albumin and apolipoprotein B by hepatocytes was observed; cytochrome P450 activity remained unaltered; gamma-glutamyltransferase activity in bile duct epithelial cells as well as alkaline phosphatase activity were within normal range; proliferative response to pancreatotropin was also maintained. These features render it a valuable platform for HBOs research.

## 4 Application of biliary organoids

The biliary organoids are primarily utilized for disease modeling, mechanism research, drug screening, tissue/organ repair, and other related aspects.

### 4.1 Disease model

#### 4.1.1 Biliary atresia

BA is a fibroinflammatory disease characterized by congenital intrahepatic and extrahepatic biliary obstruction, leading to impaired bile discharge and subsequent liver damage. It can progress to cirrhosis and liver failure, posing a life-threatening risk to children (Zhu et al., 2023). The pathogenesis of BA-induced liver fibrosis remains unknown; however, studies have suggested potential associations with functional abnormalities such as activation of the TWEAK/FN14 signaling pathway, epithelial-mesenchymal transition (EMT), β-amyloid beta (Aβ) accumulation, matrix collagen deposition, apical-basal cell polarity disorder, and primary ciliary dysfunction (Deng et al., 2011; Glessner et al., 2023; Lendahl et al., 2021; Short et al., 2023). Although various methods like drug toxicant induction, surgical induction (mechanical biliary duct ligation), rotavirus induction, gene recombination are used for establishing animal models of BA disease; they have limitations including complex procedures, high costs, and long duration. Utilizing biliary organoids offers an efficient and rapid approach for establishing BA disease models.

Chen et al. (2020) successfully generated a biliary organoid using the embedding culture techniques and co-cultured it with rotavirus to establish a disease model of BA. They observed that rotavirus induced lesions in biliary cells through virus-host interaction, leading to the development of BA. Furthermore, it is suggested that inhibiting rotavirus growth and neutralizing antibodies against the rotavirus VP7 protein could be potential treatments for BA (Chen et al., 2020). Amarachintha et al. (2022) constructed BA organoids (BACOs) by embedding biliary tract cells derived from patients with BA in matrigel. Human liver tissue was minced, washed twice with ice-cold DMEM/F12 basal medium, filtered through a 0.1 mm filter membrane, mixed with a low growth factor matrix glue, and then transferred into a 24-well culture plate with 50 uL per well. The plate was incubated at 37°C for 10 min before adding 50 uL of separation medium for culture. On the fifth day, BACOs were prepared by supplementing them with B27 (1x), N2 (1x), R-spondin 1, A83-01, vitamin A, N-acetylcysteine, nicotinamide, recombinant human gastrin, recombinant human EGF, recombinant human FGF10, recombinant human HGF, and forskolin. They found significantly decreased expression levels of markers related to biliary tract development such as KRT7, EpCAM, aquaporin 1 (AQP1), CFTR and somatostatin receptor2(SSTR2) through immunohistochemical staining in BACOs.

#### 4.1.2 Biliary tract tumors

Biliary tract tumors typically refer to primary tumors of the biliary tract system, which are categorized as benign or malignant. The main malignant tumors of the biliary tract include gallbladder carcinoma (GBC) and cholangiocarcinoma (CCA). Currently, various disease models exist for GBC, such as 2D and 3D cell cultures, xenograft models, and 3D bio-printing models; however, there are few reports on organoid disease models for gallbladder cancer (Xing et al., 2023). Cholangiocarcinoma is a type of malignant tumor that develops in the epithelial cells of the biliary tract. Based on anatomical location, it can be classified as intrahepatic cholangiocarcinoma (ICCA), perihilar cholangiocarcinoma (PCCA), or distal cholangiocarcinoma (DCCA) (Brindley et al., 2021; Loeuillard et al., 2019a; Loeuillard et al., 2019b). Disease models for CCA are typically established in experimental animals using chemical induction, tumor transplantation, biliary stasis, parasitic infection or genetic engineering methods; however these approaches have some disadvantages such as low controllability and high cost with low success rates (Calvisi et al., 2023; Domènech Omella et al., 2023). CCA organoids retain most histological and genetic characteristics while demonstrating high stability during *in vitro* culture providing a novel approach to study biliary tract cancer (Nuciforo and Heim, 2021).

Zhou et al. (2022) minced liver specimens obtained from patients with CCA and incubated the resulting digest at 37°C for 2–5 h to obtain a suspension. This suspension was then filtered through a 100 μm nylon cell filter, followed by centrifugation at 300 to 400 RPM for 5 min. The resulting cryoprecipitate was washed in advanced DMEM/F12 (GIBCO) medium. Subsequently, it was mixed with basement membrane extract type II (BME) and seeded in 24-well plates at a density of 2,000 to 5,000 cells per well. The culture medium was supplemented with penicillin/streptomycin, glutamate, HEPES, B27 supplement, N2 supplement, N-acetyl-L-cysteine (NAC), nicotinamide, recombinant human gastrin I, recombinant human EGF, recombinant human FGF10, recombinant human HGF, forskolin, A8301, ROCK inhibitor Y27632, and dexamethasone. A CCA organoid model was successfully constructed (Zhou et al., 2022). Maier et al. (2021) reported a protocol for establishing stable CCA organoids by embedding culture techniques. The digested mixture was initially filtered through filters ranging from 40 to 100 um, followed by differential centrifugation at 200 r/min for 3 min. The isolated cells and cytokines were then seeded in matrigel. Rho-associated protein kinase (ROCK), forskolin, insulin, transferrin, and selenite were added to the culture medium to establish stable CCA organoids. These aforementioned models can be utilized for investigating the molecular mechanisms underlying CCA disease as well as translational research on this condition, providing an innovative platform for studying CCA disease (Maier et al., 2021).

#### 4.1.3 Primary sclerosing cholangitis

PSC refers to a chronic hepatobiliary disease whose origin is unknown. It is characterized by the presence of multiple strictures in both internal and external biliary tracts. ERCP angiography reveals a distinctive “dead branch” pattern that indicates chronic inflammation and progressive fibrosis within the structure of the biliary tract. Eventually, this condition may lead to cirrhosis and end-stage liver disease; some cases are also associated with biliary malignancy (Reich et al., 2021). Experimental animal models are commonly used for studying PSC through genetic engineering, chemical induction, bacterial induction or other methods. However, it can be challenging to investigate disease progression under different external environments; nonetheless, organoid disease models effectively address this limitation.

Zhang et al. (2023) presented a method for constructing PSC organoids, in which PSC liver tissue was initially fragmented into small pieces (<3 mm). Subsequently, the tissue was incubated with collagenase-containing buffer for 30–40 min, followed by centrifugation of the resulting suspension at 44°C and 200 r/min for 3 min. The cell precipitation was then washed thrice with minimal essential medium to resuspend it. Fluorescent-activated cell sorter (FACS) was employed to enrich extrahepatic and intrahepatic biliary cells. After positive immunostaining of cytokeratin (CK)19, the cells were cultured in Dulbecco’s modified Eagle’s medium/F12 medium (1:1) supplemented with fetal bovine serum (10%), penicillin-streptomycin (1%), Adenine (100 umol/L), insulin (5 ug/mL), epinephrine (1 ug/mL), T3-T transferrin (8 ug/mL), epidermal growth factor (10 ng/mL), and hydrocortisone (600 ng/mL) to establish a PSA organoid (Zhang et al., 2023). This novel *in vitro* disease model of PSA provides an opportunity to investigate the pathophysiology of PSC as well as potential treatment options. Du et al. (2023) on the other hand, developed a vascularized bile duct on-a-chip (PVBDOC)-based PSC organoid using organ chip technology. The expression profile of cytokines such as biliary cell markers, polarity markers, collagen IV, laminin, bile salt transporter ASBT, secretin receptor SCTR and tight junction component ZO-1 in this disease model is consistent with that observed in biliary cells of PSC; thus making it an advantageous tool for studying both physiological and pathological mechanisms underlying PSC (Du et al., 2023). In recent years, Jalan-Sakrikar et al. (2022) reprogrammed fibroblasts from patients with PSC to obtain hiPSCs which were subsequently cultured in three dimensions leading to formation of a PSC organoid. They utilized this advanced model system effectively elucidating the pathological structure and characteristics associated with PSC thereby highlighting its immense potential as a valuab. In summary, biliary organoids can more accurately reflect the complexity and dynamic changes of the disease, providing a solid platform for disease modeling research on primary sclerosing cholangitis. Through in-depth investigation into the disease mechanism and drug screening for primary sclerosing cholangitis, it is expected to offer novel approaches for future treatment and prevention of the disease.

### 4.2 Research on disease mechanism

Biliary tract diseases impact the structure of the biliary tract through various pathophysiological mechanisms. However, current 2D cell models fail to accurately replicate the intricate architecture and precise cellular localization within the biliary tract. Moreover, controlling the size of the biliary tract and positioning cells is challenging using these models. In contrast, organoids possess distinctive spatial organization and cell specificity, making them capable of achieving tissue regeneration and restoring a majority of original organ functions. Consequently, they serve as an ideal research platform for investigating both physiological and pathological mechanisms underlying biliary tract disorders.

Jalan-Sakrikar et al. (2022) reprogrammed fibroblasts from patients with PSC to generate hiPSCs, which were then utilized to construct a biliary organoid through three-dimensional culture. By employing electron microscopy, they investigated the anatomical features of the organoid and observed its small size, absence of a central lumen, and accelerated aging. Furthermore, they identified disease-specific characteristics of PSC by detecting an upregulation in the secretion of extracellular matrix molecules (fibronectin), inflammatory cytokine IL-6, and C-C motif chemokine ligand 2. Amarachintha et al. (2022) successfully generated BACOs using liver tissue obtained from infants. The researchers employed transmission electron microscopy (TEM) to examine the physiological and anatomical structure of BACOs and noted a limited number of ciliated cells exhibiting lateral growth of cilia, potentially attributed to reduced levels of F-actin, β-catenin, and ezrin protein secretion (Amarachintha et al., 2022). In addition, decreased expression of tight junction protein zonula occludens-1 (ZO-1) was observed in biliary epithelial cells within BACOs leading to compromised barrier function and increased permeability. Moreover, activation of the EGF/FGFs signal transduction pathway was demonstrated to enhance epithelial cell differentiation as well as improve biliary epithelial barrier function in these cholangiocyte organoids (Amarachintha et al., 2022). The cystic fibrosis ECOs established by Verstegen et al. (2020) demonstrated normal chloride channel and MDR1 transporter activity, but showed a loss of CFTR channel activity. These studies underscore the crucial role played by biliary organoids as a visualization platform for investigating internal metabolism and regulatory mechanisms within the biliary system.

### 4.3 Drug screening

Currently, the commonly employed drug screening models primarily consist of two-dimensional cell models and human tumor xenograft models. However, these disease models exhibit limited accuracy in assessing drug efficacy due to disparities between the culture environment and *in vivo* conditions, as well as physiological variations across species. Organoids can effectively mimic drug sensitivity and resistance patterns within solid tissues, offering a short preparation cycle and stable passaging capability. Consequently, they hold significant potential for high-throughput drug screening (Magré et al., 2023; Yang and Yu, 2023).

Utilizing embedding culture technique, Yuan et al. (2022) successfully generated a GBC organoid and employed it to evaluate the therapeutic efficacy of CUDC-907, a dual PI3K/HDAC inhibitor, against GBC. The results demonstrated significant inhibition of GBC growth by CUDC-907 with minimal toxicity towards normal biliary epithelial cells compared to other anticancer drugs. These findings underscore the potential of biliary organoids as drug screening platforms. Ren et al. (2023) also established an organoid model for patients with CCA using embedding culture technology with a high success rate. Researchers evaluated and documented the therapeutic effects of seven commonly used chemotherapy drugs, including gemcitabine, cisplatin, capecitabine/5-fluorouracil (5-FU), SN-38 (the active metabolite of irinotecan), oxaliplatin, mitomycin C, and paclitaxel on this model. Subsequently, they compared these results with follow-up outcomes in CCA patients as well as therapeutic effects observed in CCA models in mice. Consistent drug response patterns were observed between these comparisons, further validating biliary organoids as reliable and efficient platforms for drug screening. Wang et al. established an organoid model for DCCA and used it to compare the inhibitory effects of six drugs: gemcitabine, 5-fluorouracil, cisplatin, paclitaxel, infelatinib, and ivociclib (Wang et al., 2021). The results demonstrated that gemcitabine exhibited the most potent inhibitory effect on DCCA and emerged as the most effective therapeutic option. Saito et al. (2019) established a robust organoid system for CCA through drug screening, in which the antifungal drugs amorolofine and fenteconazole exhibited significant growth inhibition of CCA-derived organoids. Importantly, these drugs demonstrated minimal toxicity towards normal biliary epithelial cells. Furthermore, analysis of the gene expression profile in these organoids identified SOX2 as a potential prognostic biomarker for CCA patients, offering valuable insights into targeted gene therapy and disease prognosis.

Chen et al. (2024) established a PSC organoid and demonstrated that exosomes derived from human placenta-derived mesenchymal stem cells (Exo^MSC^) ameliorate the hypersecretory phenotype and cell-cell interactions in the hepatic Th17 microenvironment by regulating PERK/CHOP signaling, thereby reducing hepatic fibrosis in PSC. The therapeutic potential of Exo^MSC^ has been confirmed. Nakamoto et al. (2019) developed a bacteria-PSC organoid co-culture system and revealed that *Klebsiella pneumoniae* mediates hepatobiliary injury through the TH17 pathway. Furthermore, antibiotic treatment was found to improve the TH17 immune response induced by PSC-derived microbiota, providing valuable insights into the treatment strategy for PSC (Nakamoto et al., 2019). The results suggest that biliary organoids can be a powerful research tool for drug screening, as they can elucidate the molecular pathogenesis and discover biomarkers for refractory diseases. Table 2 presents organoid models and their applications in common refractory bile duct diseases.

**TABLE 2:** Organoid models and their applications in common refractory bile duct diseases.

Types of disease	Models of disease	Applications		References
BA	BA organoids	Mechanisms of diseases	The number of ciliated cells in the bile duct decreased, and the elongation of cilia may be linked to a decrease in the secretion of F-actin, β-catenin, and ezrin proteins. Moreover, PRKCD, RASA4, SFN, RASA4B et al. Identified 16 genes related to the EGFR/FGF pathway that may serve as potential targets for disease treatment	Amarachintha et al. (2022)
		Drug screening	Antiviral agents and neutralizing antibodies target the VP7 protein	Chen et al., 2020
CCA	CCA organoids	Mechanisms of diseases	SOX2 has the potential to serve as a biomarker for predicting the prognosis of CCA. Moreover, the PI3K/HDAC pathway can be considered as a target pathway for treating this disease. Furthermore, genes such as NNMT, GSTT1, SPTSSB, and RARRES1 might also be potential targets for therapeutic interventions	Saito et al., 2019, Yuan et al., 2022
		Drug screening	CUDC-907, gemcitabine, amorofene, and fenticonazole	Yuan et al., 2022, Wang et al., 2021, Saito et al., 2019
PSC	PSC organoids	Mechanisms of diseases	The PSC organoids are smaller, lack a central lumen, and age faster than normal biliary organoids. Additionally, they secrete more extracellular matrix molecules like fibronectin, the inflammatory cytokine IL-6, and C-C motif chemokine ligand 2. Moreover, histocompatibility complex class II, DQβ1 (HLA-DQB1); Histone deacetylase 7 (HDAC7); The SET domain-containing 1A and histone lysine methyltransferase (SETD1) are potential targets for disease treatment	Jalan-Sakrikar et al. (2022)
		Drug screening	Exo^MSC^, Metronidazole and Vancomycin	Nakamoto et al., 2019, Chen et al., 2024

Abbreviations: BA, biliary atresia; CCA, cholangiocarcinoma; PSC, primary sclerosing cholangitis; PRKCD, protein kinase C delta; RASA4, RAS, p21 protein activator 4; SFN, Sulforaphane; RASA4B, RAS, p21 protein activator 4B; SOX2, a transcription factor; CUDC-907, dual PI3K/HDAC, inhibitor; NNMT, n-nicotinamide methyltransferase; GSTT1, Glutathione S-transferase theta class 1; SPTSSB, serine palmitoyltransferase complex; RARRES1, Retinoic acid receptor responder 1; HLA-DQB1, histocompatibility complex class II, DQβ1; HDAC7, histone deacetylase 7; SETD1, SET, domain-containing 1A and histone lysine methyltransferase; Exo^MSC^, extracellular vesicles derived from hPMSCs.

### 4.4 Tissue/organ repair

Clinically, bile duct injury can occur following abdominal trauma, cholecystectomy, hepatectomy, or liver transplantation and may also arise as a complication of ERCP. The standard surgical treatment is typically categorized based on the severity of bile duct injury. Bile duct repair can be performed for mild injuries, while hepatobiliary anastomosis or pancreaticobiliary anastomosis is often utilized for severe injuries. Additionally, endoscopic stent placement may be employed to ensure biliary tract patency (Cho et al., 2018; de’Angelis et al., 2021; Omar et al., 2023). Biliary organoids exhibit remarkable stability and excellent biocompatibility, making them a promising alternative for the management of biliary tract injuries in the field of biliary regenerative medicine. Before initiating clinical trials in the field of biliary regenerative medicine, researchers must gather substantial evidence from preclinical investigations to ensure the safety and efficacy of biliary organoids. This involves conducting long-term observations in animal models, thoroughly assessing the origin of biological materials and cells, as well as establishing clear indications for designing clinical study protocols (Huang et al., 2021). Clinical trials should specifically focus on biliary diseases such as biliary obstruction, cholangiocarcinoma, and refractory conditions associated with bile duct dysfunction (De Assuncao et al., 2017; Tam et al., 2024). The clinical trials involving biliary organoids in regenerative medicine should adopt a randomized controlled trial (RCT) design that encompasses evaluating the design phase and collecting and analyzing data to ensure scientific validity. Furthermore, after patients receive treatment, it is essential to conduct long-term follow-up to gather data on efficacy and side effects so that these findings can contribute towards forming scientific evaluation reports that guide subsequent studies and applications (Soroka et al., 2021).

In 2017, Sampaziotis conducted an experiment using extrahepatic biliary cells to create engineered ECOs. These ECOs were transplanted under the renal capsule of immunodeficient mice and demonstrated robust growth and development, resembling a conduit structure with high similarity to the biliary tract. Furthermore, they expressed specific markers associated with biliary cells (Sampaziotis et al., 2017). Additionally, the researchers seeded ECOs onto a biodegradable scaffold, resulting in the formation of a tissue-like structure that retained key features of bile ducts. This study provides a solid foundation for regenerative medicine applications targeting the biliary tract. X Yang utilized 3D printing technology to fabricate an artificial bile duct incorporating mesenchymal stem cells (MSCs), which was subsequently transplanted into pigs while monitoring its progress through MR Imaging (Xiang et al., 2020). After 14 days, histological examination confirmed that the morphology and structure of the artificial bile duct remained normal based on HE staining. Immunohistochemical staining revealed expression of CK19 as a marker for bile ducts, indicating successful repair and regeneration of the damaged bile duct. This achievement represents an initial step towards restoring functionality in diseased or injured bile ducts. In the future, further investigation should be conducted on the application of artificial bile ducts in refractory biliary diseases such as cholangiocarcinoma and bile duct stenosis. This aims to explore the reparative and regenerative potential of artificial bile ducts across different disease conditions. In recent years, Sampaziotis et al. (2021) further advanced their research by utilizing primary human extrahepatic biliary tract cells to construct ECOs. Through single-cell RNA sequencing analysis, they discovered mechanisms involving transcriptional diversity loss and recovery within these cells (Sampaziotis et al., 2021). Moreover, in an *ex vivo* human liver model with normothermic perfusion system, they successfully implanted ECOs into injured intrahepatic biliary tracts. The transplanted biliary cells effectively modulated bile composition and pH levels while exhibiting normal physiological function (Amarachintha et al., 2022). These findings demonstrate significant potential for utilizing biliary organoids. Biliary tract organoids have demonstrated significant research potential in the field of regenerative medicine, providing novel insights and methodologies for refractory biliary tract diseases and liver-related disorders by investigating the mechanisms of bile duct injury repair.

## 5 Summary and prospect

The application of organoid technology in biliary tissue research offers a comprehensive understanding of its cellular and molecular biology, as well as the underlying mechanisms involved in pathogenesis and tumorigenesis. This emerging field holds immense potential for disease modeling, drug screening, regenerative medicine, and investigating physiological and pathological processes. However, it is important to acknowledge that this technology is still in its early stages with certain limitations. Firstly, biliary organoids currently lack the complexity observed in human organs. Additionally, being an *in vitro* model of the biliary tract restricts their ability to study multi-organ system interactions within the human body, limiting their applicability for exploring physiological and pathological mechanisms comprehensively. Secondly, biliary organoids primarily focus on mimicking only the epithelial component of the organ while lacking crucial physiological conditions such as vascular systems, immune environments, tumor microenvironments (TME), resulting in low long-term cell and tissue survival rates. Consequently, they fail to fully replicate the intricate complexities and cell-to-cell interactions found within natural tissues. Lastly but importantly, there remains a lack of standardization regarding construction methods for biliary organoids. Current research mainly focuses on cultivating these organoids from different species without clear guidelines or regulations concerning size, shape or gene expression levels - leading to high variability among generated organoids which poses challenges during passage procedures and high-throughput drug screening applications; ultimately impeding efficient research progress and clinical translation (Hu and Lazar, 2022).

Future research should prioritize the following aspects: standardizing culture procedures and implementing unified quality control standards to enhance the consistency and reproducibility of organoid construction (Hu et al., 2023). In terms of standardized culture procedures for biliary tract organoids, it is essential for researchers to clearly specify the types of seed cells involved, such as biliary epithelial cells, iPSCs, and hepatic progenitor cells. Additionally, they should establish standardized protocols for cell acquisition and processing. Furthermore, appropriate three-dimensional culture technologies like embedding culture technology, rotating culture technology, and organ chip culture technology should be selected. The parameters of the cell culture environment need to be precisely defined and a detailed schedule for the entire culture process should be formulated. Moreover, methods need to be developed in order to assess the functionality of biliary organoids including bile secretion capacity, cell proliferation rate, and multilineage differentiation potential. Quality control standards must also be established through regular evaluations of both the quality of cells used and the composition of the culture medium employed. Standardization in all aspects of culturing operations is crucial, while specific verification criteria should be devised for biliary tract organoids. Furthermore, continuous evaluation of both cultural effects and quality control measures is necessary to ensure that final biliary tract organoids possess desirable biological characteristics. Lastly but importantly, establishing a comprehensive database dedicated solely to biliary organoids would provide invaluable data support for future research endeavors while simultaneously promoting their application and development. Utilizing bioreactors can improve nutrient transportation and metabolic waste removal in organoids, thereby extending their lifespan (Rimal et al., 2024). Co-culturing seed cells with vascular endothelial cells or their progenitors can promote angiogenesis and increase organoid survival rate (Rimal et al., 2024). Organ-on-a-chip technology enables the cultivation of 3D vascularized organoids, facilitating flux generation, vascularization formation, interaction between organoids, and control over tissue microenvironment. This advancement is crucial for guiding stem cell growth, differentiation, morphogenesis of organoids, as well as overcoming existing limitations in this field of research (Du et al., 2020; Monteduro et al., 2023). The co-cultivation of seed cells and mesenchymal cells can enhance the differentiation and maturation of biliary organoids. Additionally, supplementing the medium with R-spondin and DKK-1 can provide paracrine signals to induce cell differentiation and facilitate the formation of a three-dimensional structure in biliary organoids (Ock et al., 2023). With continuous advancements in organoid technology, biliary tract organoids will play an increasingly significant role in related fields.
