# Supplementary figures and images for: The utilisation of biliary organoids for biomedical applications
Source: Front Bioeng Biotechnol. 2025 Jan 7;12:1501829. doi: 10.3389/fbioe.2024.1501829 (PMC11753252; doi:10.3389/fbioe.2024.1501829)

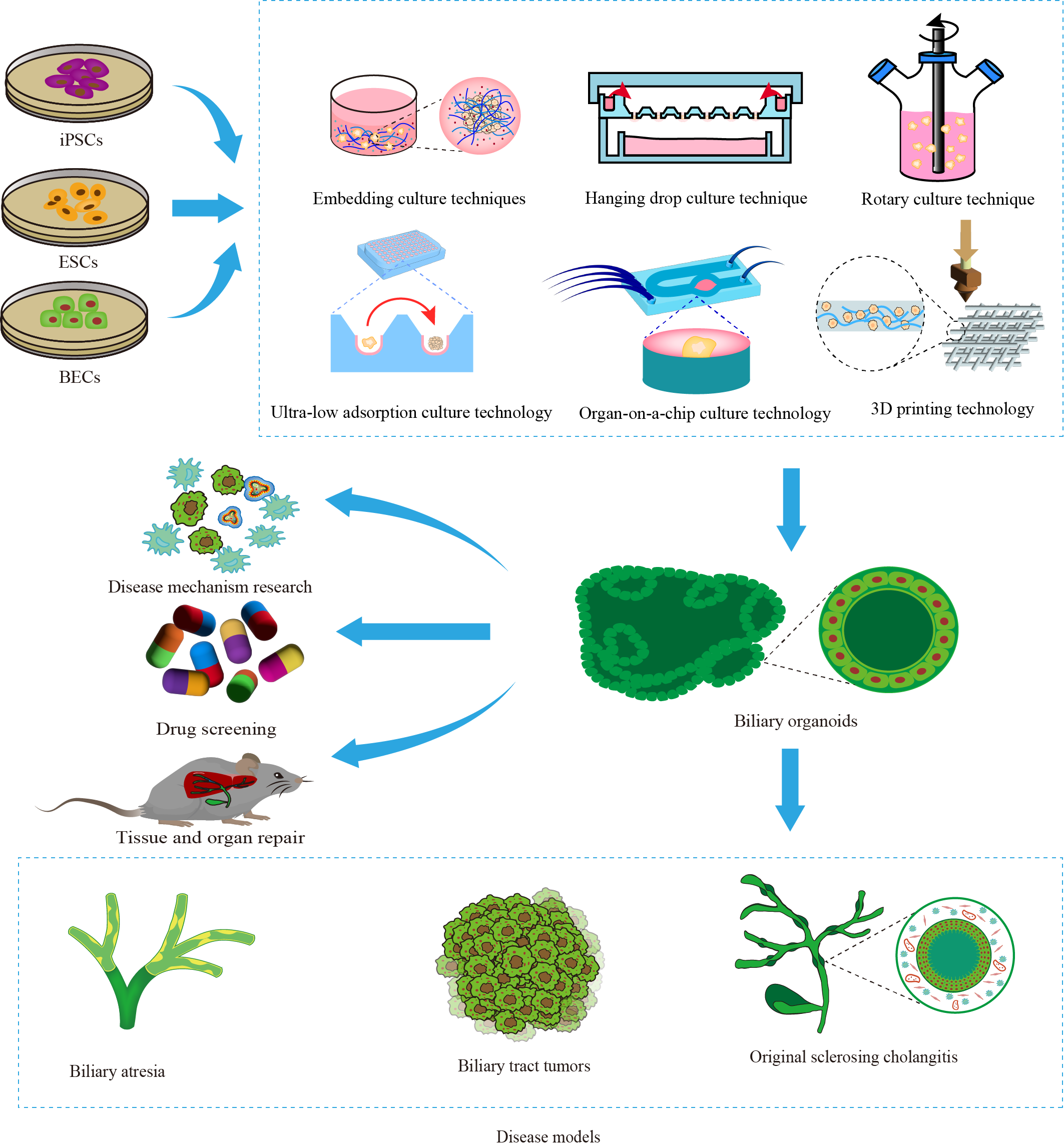

Supplement: Supplementary file 1 [file DataSheet1.ZIP › Figure.1 Construction and application of biliary organoids..tif]

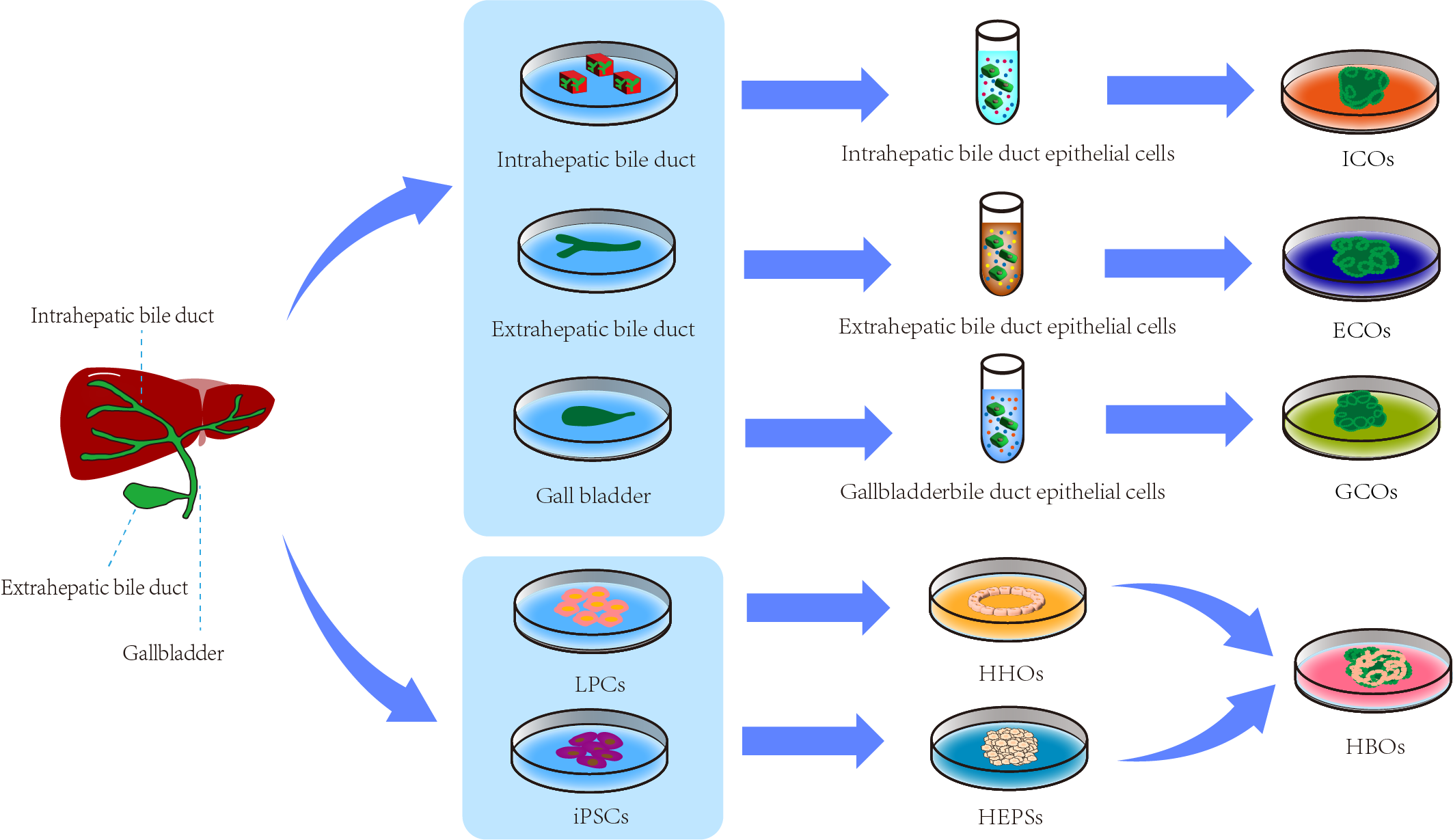

Supplement: Supplementary file 1 [file DataSheet1.ZIP › Figure.2 Types of biliary organoids..tif]
