# Supplementary material for: The utilisation of biliary organoids for biomedical applications
Source: Front Bioeng Biotechnol. 2025 Jan 7;12:1501829. doi: 10.3389/fbioe.2024.1501829 (PMC11753252; doi:10.3389/fbioe.2024.1501829)
Supplement: Supplementary file 2 [file DataSheet2.ZIP › Supplementary material presentation/Table.docx]

**Table 1 Several common organ construction techniques**

| Technology of construction | Type of technology | Advantages | Disadvantages | Type | References |
| --- | --- | --- | --- | --- | --- |
| Traditional techniques | Embedding culture techniques | The procedure is simple, demonstrating strong biocompatibility and the ability to control the porosity of the outer wall | The lack of standardization in the matrix, which typically undergoes compositional changes between batches, leads to significant variations in the generated organoids. | ICOs, ECOs, GCOs, HBOs | S. Y. Lee *et al.*, 2021, Lugli *et al.*, 2016, Sampaziotis *et al.*, 2017, Ramli *et al.*, 2020, Amarachintha *et al.*, 2022 |
|  | Rotary culture technique | It is capable of simulating the microgravity effect and exhibits a high rate of nutrient utilization. | It requires a high rotation rate and expensive equipment | HBOs | Ryu,Lee and Park, 2019, He *et al.*, 2022 |
|  | Hanging drop culture technique | The procedure is simple, the size of the organoids remains consistent, and there is a significant amount of flux | The stability of organoids is suboptimal, and replacing the culture medium presents challenges | Tubuloids，TOs, MOs | S. Zhou *et al.*, 2023, Parigoris *et al.*, 2022, Y. Yang *et al.*, 2022 |
|  | Magnetic suspension culture technology | It facilitates the co-cultivation of multiple cells, enhances cellular interaction forces, and promotes cell aggregation | The equipment is expensive, and controlling the size of cell spheres presents challenges | SGLOs, AOs | Tepe,Aslanbay Guler and Imamoglu, 2023 Daquinag,Souza and Kolonin, 2013, Ferreira *et al.*, 2019 |
|  | Ultra-low adsorption culture technology | It is easy to operate, manageable, and has a high processing capacity | The high coefficient of variation in organoids and the ultra-low adsorption culture plates come at a significant cost | HBOs | Ryu,Lee and Park, 2019, Kim *et al.*, 2023, Acharya *et al.*, 2024 |
| New technologies | Organ-on-a-chip culture technology | It allows for three-dimensional dynamic cultivation, precise control of physical and chemical stimuli, as well as high-throughput and reliable operations | When certain materials, such as PDMS, are used, the drugs tested in the system exhibit low permeability | BOs | Baptista *et al.*, 2024, Palasantzas *et al.*, 2023, Du *et al.*, 2020 Grant et al., 2021 |
|  | 3D printing technology | It offers the benefits of personalized customization, a high level of refinement, and rapid modeling speed | The lack of legal frameworks, regulatory measures, and established quality benchmarks is evident | HBOs | Mandal and Chatterjee, 2024, Assad,Assad and Kumar, 2023, Jing *et al.*, 2023, H. Lee *et al.*, 2019 |
|  | 4D printing technology | The deformation structure can be controlled, the stimulus response types are diverse, and the strain recovery is reversible | The response form is singular, and there is a scarcity of high-performance intelligent materials | GPDOs | Kalogeropoulou *et al.*, 2024, Wan *et al.*, 2024, Chadwick *et al.*, 2020, Y. Li *et al.*, 2024b |

Abbreviations: PDMS, polydimethylsiloxane; ICOs, intrahepatic biliary organoids; ECOs, Extrahepatic cholangiocyte organoids; GCOs, gallbladder biliary organoids; HBOs, hepatobiliary organoids; Tubuloids, basal-in and apical-out proximal tubule organoids; TOs, testicular organoids; MOs, mammary organoids; SGLOs, salivary gland-like organoids; Aos, adipose organoids; BOs, biliary organoids; GPDOs, glioblastoma patient derived organoids-like.

**Table 2 Organoid models and their applications in common refractory bile duct diseases**

| Types of disease | Models of disease | applications | | References |  |
| --- | --- | --- | --- | --- | --- |
| BA | BA organoids | Mechanisms of diseases | The number of ciliated cells in the bile duct decreased, and the elongation of cilia may be linked to a decrease in the secretion of F-actin, β-catenin, and ezrin proteins. Moreover, PRKCD, RASA4, SFN, RASA4B et al. identified 16 genes related to the EGFR/FGF pathway that may serve as potential targets for disease treatment. | Amarachintha *et al.*, 2022 |  |
|  |  | Drug screening | Antiviral agents and neutralizing antibodies target the VP7 protein | S. Chen *et al.*, 2020 |  |
| CCA | CCA organoids | Mechanisms of diseases | SOX2 has the potential to serve as a biomarker for predicting the prognosis of CCA. Moreover, the PI3K/HDAC pathway can be considered as a target pathway for treating this disease. Furthermore, genes such as NNMT, GSTT1, SPTSSB, and RARRES1 might also be potential targets for therapeutic interventions. | Saito *et al.*, 2019，Yuan et al., 2022 |  |
|  |  | Drug screening | CUDC-907, gemcitabine, amorofene, and fenticonazole | Yuan *et al.*, 2022, Z. Wang *et al.*, 2021,Saito *et al.*, 2019 |  |
| PSC | | PSC organoids | Mechanisms of diseases | The PSC organoids are smaller, lack a central lumen, and age faster than normal biliary organoids. Additionally, they secrete more extracellular matrix molecules like fibronectin, the inflammatory cytokine IL-6, and C-C motif chemokine ligand 2. Moreover, histocompatibility complex class II, DQβ1 (HLA-DQB1); Histone deacetylase 7 (HDAC7); The SET domain-containing 1A and histone lysine methyltransferase (SETD1) are potential targets for disease treatment. | Jalan-Sakrikar *et al.*, 2022 |
|  |  |  | Drug screening | Exo^MSC^, Metronidazole and Vancomycin | Nakamoto *et al.*, 2019, W. Chen *et al.*, 2024 |

Abbreviations: BA, biliary atresia; CCA, cholangiocarcinoma; PSC, primary sclerosing cholangitis; PRKCD, protein kinase C delta; RASA4, RAS p21 protein activator 4; SFN, Sulforaphane; RASA4B, RAS p21 protein activator 4B; SOX2, a transcription factor; CUDC-907, dual PI3K/HDAC inhibitor; NNMT, n-nicotinamide methyltransferase; GSTT1, Glutathione S-transferase theta class 1; SPTSSB, serine palmitoyltransferase complex; RARRES1, Retinoic acid receptor responder 1; HLA-DQB1, histocompatibility complex class II, DQβ1; HDAC7, histone deacetylase 7; SETD1, SET domain-containing 1A and histone lysine methyltransferase; Exo^MSC^, extracellular vesicles derived from hPMSCs
